# The mini-Oxford cognitive screen (Mini-OCS): A very brief cognitive screen for use in chronic stroke

**DOI:** 10.1093/esj/23969873251358811

**Published:** 2026-01-01

**Authors:** Sam S Webb, Luning Sun, Eugene Yee Hing Tang, Nele Demeyere

**Affiliations:** Nuffield Department of Clinical Neurosciences, University of Oxford, Oxford, UK; The Psychometrics Centre, University of Cambridge, Cambridge, UK; Population Health Sciences Institute, Newcastle University, Newcastle upon Tyne, UK; Nuffield Department of Clinical Neurosciences, University of Oxford, Oxford, UK

**Keywords:** Cognition, chronic stroke, psychometrics, cognitive screening

## Abstract

**Introduction:**

No stroke-specific cognitive screen currently exists for community-dwelling chronic stroke survivors, with primary care and community settings relying on dementia tools which often do not consider specific post-stroke impairments. The Oxford Cognitive Screen (OCS) was developed for use in acute stroke, but its administration time is prohibitive for brief screening. Here, we aimed to develop, standardise and psychometrically validate the Mini-Oxford Cognitive Screen (Mini-OCS), a brief (<8 min) cognitive screen aimed for use in chronic stroke.

**Method:**

Existing full OCS data for 464 English participants who were ⩾6 months post-stroke were analysed for the possibility of a short-form. Theoretical choices were made to adapt the short-form to be suitable for use in chronic stroke. The Mini-OCS was then completed by 164 neurologically healthy controls (*M*_age_ = 68.66; SD = 12.18, *M*_years_ of education 15.40; SD = 3.64, 61% female), and 89 chronic stroke survivors (*M*_age_ = 69.86; SD = 14.83, *M*_years_ education = 14.29; SD = 4.01, 44.94% female, *M*_days_ since stroke = 597.02; SD = 881.12, 78.57% ischaemic, Median NIHSS = 6.5 (IQR = 4–11)). In addition, the original OCS, the Montreal Cognitive Assessment, and an extended neuropsychological battery were administered. Psychometric properties of the Mini-OCS were evaluated via construct validity and retest reliability.

**Findings:**

Normative data for the Mini-OCS is provided and known-group discrimination demonstrates increased sensitivity in the memory and executive function domains compared to the OCS. The Mini-OCS further met all appropriate benchmarks for evidence of retest reliability and construct validity.

**Discussion and conclusion:**

The Mini-OCS is a short-form standardised cognitive screening tool with initial evidence of good psychometric properties for use in a chronic stroke population.

## Graphical Abstract

**Graphical abstract:**
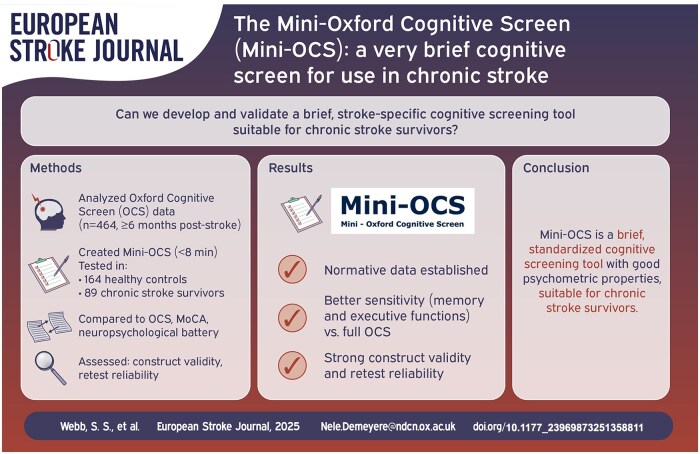

Stroke is a leading cause of disability and mortality across the globe,^1,2^ leading to high rates of cognitive impairment often affecting in excess of 85% of stroke survivors acutely after stroke, with long term prevalence of cognitive impairment estimates up to 65%.^2–4^ Currently, little attention has been paid to advise on routine cognitive screening long-term post-stroke, despite very high rates of objective and subjectively reported cognitive impairment,^3–6^ and the most frequently reported unmet patient need being managing cognition/mood post-stroke.^7^

The majority of post-stroke care sits with primary and community care services upon discharge from specialist services. Consultations in primary care are time limited and existing tools may not always pick up or take into account post-stroke sequelae. Brief cognitive screening tools are needed to detect focal impairments, alongside domain-general tests of memory and executive function, as distinctive markers of vascular degenerating cognitive health.^8^ NHS England’s National Stroke Programme reported that screening and assessment to improve access to effective treatments in relation to the psychological impact of stroke is a key evidence gap.^9^ By monitoring cognition and screening in primary care, appropriate onward referral to specialist settings (e.g. memory clinics, neuropsychological services) can be made in a timely manner so that the stroke-survivor and their families can gain access to potential symptomatic treatment (for cognitive impairments), neuropsychological formulation and support. Early identification of declining cognition will also mean that patients can access disease modifying treatment when this is available.

In community-based primary care settings, common assessments include the Montreal Cognitive Assessment (MoCA), General Practitioner assessment of Cognition (GPCOG),^10^ and 6-Item Cognitive Impairment Test (6CIT),^11^ which are all designed for dementia. Post-stroke cognitive profiles differ from dementia profiles, with key domains (neglect, apraxia, reading, writing etc.) not assessed in dementia screening. Further, the verbal nature of the tests (e.g. intact expressive language needed for 25/30 points of the MoCA)^12^ mean these are prone to confounding memory with language impairments where present (particularly after left hemisphere stroke). The Oxford Cognitive Screen (OCS)^13^ provides a multi-domain, stroke specific screen, and was designed as a first-line screening tool to determine the extent of focal cognitive deficits incurred by the stroke. The OCS has seen a wide take up in acute stroke-specific settings. Its length (approx. 20 min) and focus on acute deficits such as hemispatial neglect, apraxia, reading/writing impairments, as well as its aphasia adaptations, means it works well as a first line screening tool to detect the immediate effects of the stroke, but it is less sensitive at detecting domain-general deficits in memory and executive function,^14^ which are often present long-term. The OCS-Plus^15,16^ was designed as a highly sensitive test for these domain-general impairments in memory and executive function, but its additional duration and focus on milder deficits makes it impractical in time-pressured settings. Both screening tools combined would take approximately 40 min to complete. Even separately, the OCS nor the OCS-Plus are brief enough to fit within a brief appointment in primary care, and thus would never be considered for administration in these settings. A compromise solution where separate domains are briefly assessed, alongside sensitive screening for memory and executive impairments, fitting within the very short appointments in primary care is called for.

Here we set out to design a brief and sensitive cognitive screen following practical, theoretical, and statistical considerations, incorporating OCS and OCS-Plus tasks: the Mini-OCS. The current study aimed to develop the Mini-OCS, then standardize and validate the brief test psychometrically in stroke.

## Methods

The study adheres to the COSMIN guideline for studies on measurement properties^17^ and the STROBE cohort checklist.^18^

### Ethical approval procedures

Ethical approval for the study was granted by the Medical Sciences Interdivisional Research Ethics Committee at the University of Oxford (REF: R86339/RE001).

### Development

The practical development considerations for the Mini-OCS were that the test should be able to fit into a 10-min time slot, and that it required as little paperwork as possible, to reduce key barriers for uptake in primary care settings.^19^ Theoretical considerations were that the test should cover all cognitive domains commonly affected post-stroke, such as language, memory, attention, praxis, numerical processing and executive function; and it should do so in a way to detect more mild impairments which may be otherwise hidden in chronic stroke survivors. By retaining the domains, and providing domain-specific normative cut-offs, we aimed to preserve the domain-specificity of the OCS, including its ability to detect strengths (performance within norm range) as well as weaknesses (performance below norm range) in a patient’s cognitive profile from this screen. Statistical considerations were that the test must cover the domains of cognition and not constrain cognition to a single metric, where impairment is multi-facetted and multi-dimensional.^20,21^ We used classical test theory and Item Response Theory to develop a short-form OCS based on existing full OCS data from 464 participants, and create the Mini-OCS. This development is extensively described in the Supplemental Materials for manuscript brevity. Essential steps included IRT modelling of the OCS to generate a short-form that correlated highly with the full-form, then adding in tasks from the OCS-Plus to increase sensitivity to memory and executive function impairments. We iteratively developed and piloted the tasks, details of which are also in the Supplemental Materials.

### Normative and psychometric study

#### Participants

Neurologically healthy adults were recruited, either from our healthy ageing research volunteer database, or as family/friends/partners of the stroke survivors in the study, or through demographically targeted advertising on social media (Facebook). The following inclusion criteria applied: (1) no self-reported neurological or psychiatric complaints or diagnoses; and (2) a Montreal Cognitive Assessment (MoCA) score > 22^22^ on the day of participation. Note, this reduced cut-off was used in line with the recommended adjusted cut-off for stroke,^23^ and the inclusion of older adults where the original cut-off may be too strict.^24^

Chronic stroke survivors (at least ~6 months post-stroke) were recruited from a research stroke volunteer database held by the Oxford Translational Neuropsychology research group. All stroke survivors were based in the community at time of recruitment and were at least 6 months post stroke. The following inclusion criteria applied: (1) confirmed clinical stroke diagnosis from medical notes, (2) able to concentrate for at least 20 min (judged by the participant), and (3) able to give informed consent (mental capacity assessed as part of consent process following approved protocol).

Exclusion criteria for both groups included sensory/perceptual/motor impairments that would prevent the ability to complete the tasks beyond reasonable adjustment (not inclusive of wheelchair/assistance use, which still allowed participants to complete the tasks). Part way through normative participant recruitment, we restricted recruitment of healthy participants to those with equal to or less than 13 years of education, to better match the stroke population.

A priori power calculations indicated a minimum of 182 participants for the convergent/discriminant validity correlation analyses (alpha = 0.05/24 (where 24 are all 12 Mini-OCS tasks analyses at least twice – Bonferroni correction) across all sample groups, power = 90%, one-sided, correlation > 0.30). No power analysis can be conducted for determining normative sample size though we aimed to collect at least 100, but continued beyond this to include additional participants with low education (<12 years) to ensure a representative sample.

#### Measures

We administered the MoCA (the most commonly used tool across acute and community settings) and a brief battery of neuropsychological assessments. All selected tests have evidence of validity for stroke, including: the original Oxford Cognitive Screen version A^13^; the Comprehensive Aphasia Test (CAT) battery number multiple-choice number calculations^25,26^; the Boston Diagnostic Aphasia Examination pretend objects and 10 sentence reading subtasks^27,28^; The Cognitive Linguistic Quick Test symbol trails^29^; and the Behavioral Inattention Test star cancellation.^30^ Stroke severity was established via acute National Institute of Stroke Scale (NIHSS)^31^ scores from medical notes. Discriminant validity was assessed by comparison of the Mini-OCS tasks to OCS praxis, OCS orientation, and OCS cancellation accuracy where appropriate (e.g. we did not compare cancellation accuracy on Mini-OCS to OCS cancellation for discrimination). Administration time for the MoCA and Mini-OCS were recorded for comparison.

#### Data analysis

Participant demographics, new normative data, and retest consistency and convergence and discrimination were examined. We note here that the range of Mini-OCS tasks precluded the use of continuous variable intraclass correlation coefficients (accounting for practice effects). Instead, an ANCOVA examining performance scores by time point (test or retest) and controlling for change in MoCA score across time was used to detect test-retest differences. Convergent and discriminant validity was assessed against the MoCA as a reference standard for chronic stroke cognitive screening, and against the neuropsychological battery and OCS, using correlational analyses in the full mixed sample. The benchmark for convergent/discriminant validity was determined as *r*> (or< for discriminant validity) 0.30.^16,23,32^ A total score for the Mini-OCS was generated which used the regression method to calculate factor scores on the basis of a unidimensional confirmatory factor analysis (maximum likelihood estimator) using raw subtask scores from the Mini-OCS that controlled for covariance of within subtasks scores (e.g. the broken hearts tests had 3 metrics and these were allowed to co-vary).

##### Statistical analysis software

All statistical analysis and data wrangling was computed in R Studio version 4.0.4.^33^ We used the following additional packages for the production of the RMarkdown manuscript and analysis: readxl version 1.3.1^34^; cowplot version 1.1.1^35^; ggplot2 version 3.3.5^36^; kableExtra version 1.3.4^37^; ggpubr version 0.6.0^38^; and tidyr 1.2.0^39^. For statistical analyses, lavaan version 0.6.12^40^, psych version 2.4.3^41^, and catR version 3.17^42^ were used. Data and analysis scripts to recreate the manuscript are openly available in CC-BY 4.0 license (https://doi.org/10.17605/OSF.IO/CE3ZS).

All materials are available through Oxford University Innovations, who hold the copyright, with licences to be provided free of charge for publicly funded research and clinical use, in line with the approach taken for the Oxford Cognitive Screen (standard version).

## Results

### Participants

**Figure 1.**
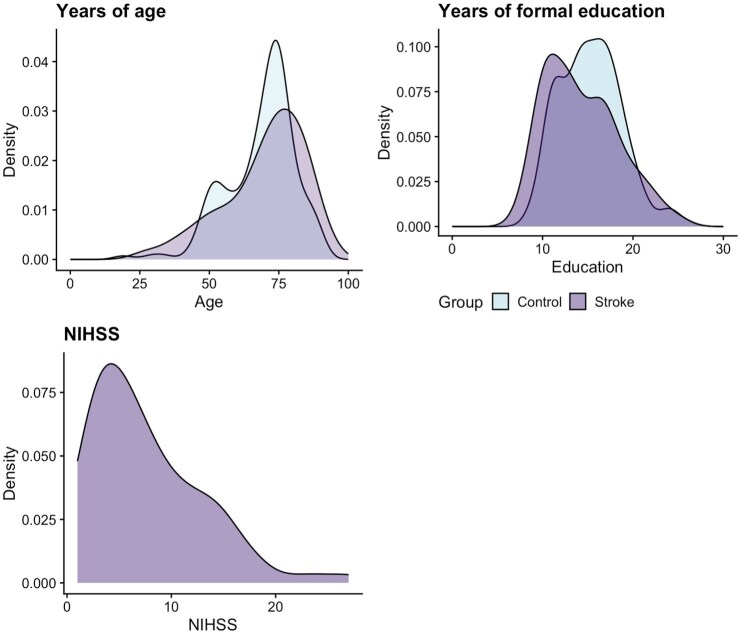
Density distribution of key sample characteristics (for both the normative and stroke cohort sample) of age, education and stroke severity (for the stroke sample only) via the National Institute of Health Stroke Scale (NIHSS).

In total, 164 neurologically healthy adults (*N* = 174 recruited, with 10 excluded for scoring below 23 on the MoCA) and 89 stroke survivors completed the Mini-OCS. All demographics are reported in Table 1 and Figure 1, which presents a visualisation of key variables for the sample, including the overlap of age and education for the two samples, and the distribution of stroke severity scores (acute NIHSS^31^) for stroke survivors. Sample groups were statistically different in years of education (mean 15.4 vs 14.4 years – *t*(166.45) = 2.18, *p* = 0.03, *d* = −0.3), but not in age *t*(153.12) = −0.65, *p* = 0.52, *d* = 0.09).

**Table 1.** Summary of sample characteristics for all participants.

Demographic	Controls		Stroke	
	*N* (missing %)	Value	*N* (missing %)	Value
Age (*M*(SD))	164 (0%)	68.66 (12.18)	89 (0%)	69.86 (14.83)
Education (*M*(SD))	164 (0%)	15.4 (3.64)	89 (0%)	14.29 (4.01)
Handedness	164 (0%)	*R* = 89.02%, *L* = 10.37%, *B* = 0.61%	89 (0%)	*R* = 85.39%, *L* = 12.36%, *A* = 1.12%, *B* = 1.12%
Sex	164 (0%)	*F* = 64.63%, *M* = 35.37%	89 (0%)	*M* = 55.06%, *F* = 44.94%
Ethnicity	164 (0%)	White: English, Welsh, Scottish, Northern Irish or British = 85.98%, White: Any other white background = 9.15%, White: Irish = 1.22%, Asian or Asian British: Chinese = 0.61%, Asian or Asian British: Indian = 0.61%, Black, Black British, Caribbean, or African: Caribbean = 0.61%, Mixed or multiple ethnic groups: Any other Mixed or multiple ethnic group background = 0.61%, Mixed or multiple ethnic groups: White and Asian = 0.61%, White: Roma = 0.61%	89 (0%)	White: English, Welsh, Scottish, Northern Irish or British = 91.01%, Other ethnic group: Any other ethnic group = 2.25%, Asian or Asian British: Any other Asian background = 1.12%, Asian or Asian British: Indian = 1.12%, Black, Black British, Caribbean or African: Any other Black, Black British, Caribbean, or African background = 1.12%, Black, Black British, Caribbean or African: Caribbean = 1.12%, White-British = 1.12%, White: Any other white background = 1.12%
Days since stroke (*M*(SD))	—	—	84 (6%)	597.02 (881.12, 167–4472)
Stroke type	—	—	84 (8.38%)	Ischaemic = 78.57%, intracerebral haemorrhage = 15.47%, multiple = 2.38%, subarachnoid haemorrhage = 1.19%
Stroke side	—	—	84 (6%)	*R* = 48.81%, *L* = 40.48%, *B* = 5.95%
Stroke severity (median (IQR))	—	—	84 (6%)	6.50 (4–11)
Modified Rankin Scale (mRS)	158 (4%)	0 = 94.94%, 1 = 3.16%, 2 = 1.9%	80 (10%)	0 = 13.75%, 1 = 18.75%, 2 = 18.75%, 3 = 36.25%, 4 = 6.25%, 5 = 6.25%

Missing data is presented in parentheses as a percentage next to *N* per demographic. Stroke severity is established via the National Institute of Health Stroke Scale. We used ethnic categories devised by the UK government census data 2021.

### Normative data for Mini-OCS

The Mini-OCS took on average 7.33 min to complete (SD = 1.63, range = 4–13 min) for controls, whereas stroke survivors were slower and took on average 8.70 min (SD = 2.63; Welch Two Sample *t*-test, *t*(109.56) = 4.27, *p* < 0.001, 95% CI: 44–120.24).

Fifth centile cut-offs for accuracy scores (95th centile for error scores) are presented for all subtasks with limited ranges of scores, whilst the executive function trail making task, the cancellation accuracy, and time to complete the Mini-OCS, employed a 1.65 SD *based* cut-off, as in the OCS-Plus.^15^ The normative data cut-offs are presented in Table 2, including a stratification by age. Age brackets were chosen by selecting the most equal segregation of age groups using the ‘split’ function in *r*.

**Table 2.** Normative data for the Mini-OCS including 5th and 95th centile cut offs, and for wider range tasks, 1.65 SD based cut offs for classifying possible cognitive impairment per task, further stratified by age classification grouping.

Measure	All neurologically healthy aging adults							<68 years			68–76 years			>76 years		
	*N*	Mean (SD)	Min	Max	5th	95th	1.65 SD	5th	95th	1.65 SD	5th	95th	1.65 SD	5th	95th	1.65 SD
Orientation	164	3.99 (0.11)	3	4	<4			<4			<4			<4		
Number calculations	123	3.76 (0.45)	2	4	<3			<3			<3			<3		
Immediate recall 1	164	4.41 (0.85)	0	5	<3			<**2**			**<4**			<3		
Immediate recall 2	164	4.90 (0.38)	2	5	<4			<**5**			<4			<4		
Meaningless gesture imitation (praxis)	164	1.87 (0.4)	0	2	<1			<1			<1			<1		
Broken hearts total	164	28.6 (2.25)	10	30	<25		<25	<**24**		<**24**	**<27**		<**27**	<25		<25
Broken hearts allocentric neglect	164	0.02 (0.22)	−1	1	<0	>0		<**−2**	>**3**		<**−1**	>**2**		<0	>2	
Broken hearts egocentric neglect	164	0.29 (1.19)	−6	4	<−1	>2		<**0**	>**0**		<**0**	>**0**		<**0**	>**0**	
Executive function	123	11.56 (4.03)	0	14			<5			<5			<**7**			<**2**
Delayed recall	164	3.85 (1.35)	0	5	<1			<**2**			<**1**			<**0**		
Sentence reading	164	14.68 (0.61)	12	15	<13			<**14**			<13			>**14**		
Time (seconds)	164	439.88 (97.33)	240	780		>600	>293.88		>600	**>284.00**		>**540**	>**275.86**		>**678**	>**317.42**
Mini-OCS total score	164	0 (0.27)	−1.91	0.17	−0.28		−0.45	−0.24		−0.35	−0.34		−0.6	−0.28		−0.25

The cut off for executive function used the 1.65SD based cut, the rest of the tasks used 5th centile in general or both 5th and 95th centile for allo- or ego-centric neglect. Normative data for the trails and number calculation tasks were taken only from Mini-OCS version 1.4.1 onwards – see Supplemental data for details. Differences in age group cut offs from the overall control sample are noted with bold cut offs. All values are rounded to nearest whole number except for time.

Performance on the Mini-OCS subtasks with a restricted range of outcome scores (e.g., orientation, number calculations, allo- and ego-centric neglect) were not found to differ significantly between age and education groups (Table S3).

### Psychometric evidence

Extensive psychometric reliability and validity evidence for the Mini-OCS is presented in full in the Supplemental materials. In brief, we found no differences in Mini-OCS performance across test and retest. For convergent validity, all Mini-OCS scores correlated with at least one matched task per comparison (e.g. some metrics used more than one matched task) above a pre-defined benchmark of *r* = 0.30, except broken hearts ego- and allo-centric neglect scores, likely due to very little spatial neglect present in this chronic sample. For discriminant validity, no correlations exceeded the benchmark of *r* = 0.30, except immediate recall 1 and OCS praxis (*r* = 0.31).

We note some differences between associations with OCS and those with neuropsychological subtests. For instance, whilst the Mini-OCS orientation subtest was significantly associated with both MoCA orientation and OCS orientation subtests, only the association with OCS orientation reached the pre-defined benchmark of *r* =0.30, likely due to MoCA orientation questions including more difficult items such as exact date.

The model fit for the total score of the Mini-OCS was acceptable; *x*^2^(41) = 44.93, *p* = 0.31, CFI = 0.92, TLI = 0.89, RMSEA = 0.02, SRMR = 0.06, suggesting a unidimensional model fits the current data. The predicted factor scores are referred to as ‘Mini-OCS total score’ henceforth.

Finally, to aid interpretation of impairment on the Mini-OCS, we examined sample differences in Mini-OCS performance, and how many stroke survivors were classified as impaired on the Mini-OCS. Note, given the heterogeneity of stroke, many stroke survivors are expected to fall in the normal range, and do not present with cognitive impairment. Mini-OCS performance scores were able to differentiate stroke survivors from neurologically healthy controls even when accounting for education effects, with overall 7.9% of chronic stroke survivors in this sample showing an impairment. The results are presented in Table 3. Notably, the higher rates of impairment were found in the memory and executive function subtasks, which is in line with our inclusion of the more sensitive memory and executive function subtasks taken from the OCS-Plus (8.30%–6.32% for immediate recall 1 and 2, and 10.28% for executive function).

**Table 3.** Descriptive statistics (*M*(SD)) for neurologically healthy ageing controls and stroke survivors on Mini-OCS metrics, with ANCOVA sample differences analyses (covarying for education differences).

Measure	Control *M*(SD)	Stroke *M*(SD)	ANCOVA	% Stroke survivors impaired
Orientation	3.99 (0.11)	3.78 (0.56)	*F*(1, 250) = 20.68, *p* < 0.001**, partial eta squared = 0.08	6.72%
Number calculations	3.76 (0.45)	3.37 (0.95)	*F*(1, 250) = 18.64, *p* < 0.001**, partial eta squared = 0.07	2.77%
Immediate recall 1	4.41 (0.85)	3.81 (1.24)	*F*(1, 250) = 18.25, *p* < 0.001**, partial eta squared = 0.07	8.30%
Immediate recall 2	4.9 (0.38)	4.31 (0.95)	*F*(1, 250) = 44, *p* < 0.001**, partial eta squared = 0.15	6.32%
Meaningless gesture imitation (praxis)	1.87 (0.40)	1.64 (0.69)	*F*(1, 250) = 9.77, *p* = 0.002**, partial eta squared = 0.04	5.93%
Broken hearts total	28.6 (2.25)	26.62 (6.03)	*F*(1, 250) = 14.44, *p* < 0.001**, partial eta squared = 0.06	11.07%
Broken hearts allocentric neglect	0.02 (0.22)	0.11 (1.05)	*F*(1, 250) = 0.56, *p* = 0.453, partial eta squared = 0	7.91%
Broken hearts egocentric neglect	0.29 (1.19)	0.13 (1.63)	*F*(1, 250) = 0.89, *p* = 0.347, partial eta squared = 0	10.67%
Executive function	11.56 (4.03)	8.99 (5.11)	*F*(1, 250) = 11.91, *p* = 0.001**, partial eta squared = 0.04	10.28%
Delayed recall	3.85 (1.35)	3.08 (1.63)	*F*(1, 250) = 13.97, *p* < 0.001**, partial eta squared = 0.05	5.53%
Sentence reading	14.68 (0.61)	13.99 (2.09)	*F*(1, 249) = 16.26, *p* < 0.001**, partial eta squared = 0.06	3.57%
Overall duration	439.88 (97.33)	522 (157.85)	*F*(1, 239) = 24.18, *p* < 0.001**, partial eta squared = 0.09	98.76%
Mini-OCS total score	0 (0.27)	−0.02 (0.27)	*F*(1, 250) = 0.36, *p* = 0.551, partial eta squared = 0	5.93%

^**^Refers to significance below an alpha corrected level of 0.05/13 = 0.003.

## Discussion

We developed a stroke-specific short-form cognitive screening tool for use with community-dwelling chronic stroke survivors: the Mini-OCS.

Following initial statistical confirmation of the possibility to shorten the OCS by half, with strong correlations between the IRT-modeled theta parameters for the full and short-form version, final development choices were based on theoretical (i.e. multi-dimensional nature of heterogeneous cognitive impairment post-stroke) and practical (i.e. suitability for primary care and community consultations and settings) considerations, resulting in a rapid screen taking less than 8 min to complete on average.

The Mini-OCS was standardised and psychometrically validated, with initial reliability and validity evidence presented alongside, in a normative and chronic stroke sample. The validity of the Mini-OCS measures was evaluated against a series of matched standard neuropsychological assessments. The Mini-OCS was found to have good convergent and discriminant validity, with the exception of allo- and ego-centric neglect measures, which lacked variance in scores due to very low levels of spatial neglect subtypes found in this chronic stroke sample. This is in line with previously reported good recovery trajectories for spatial neglect.^43,44^

Mini-OCS performance scores were able to differentiate stroke survivors from neurologically healthy controls even when accounting for education effects. Time taken to complete the Mini-OCS by stroke survivors was consistently slower than the controls, confirming an overall slowing of responses and processing speed typically found.^45,46^

### Normative data

Age adjusted cut-offs differentially affected Mini-OCS subtasks. We suggest end users refer to age adjusted cut-offs specific to Mini-OCS task in light of age-related differences. No effect of education on performance was apparent (See Table S3). This may be due to relatively limited variance in education in the sample (i.e. most participants had at least completed education to age 16), as well as perhaps due to the nature of what some of the stroke-specific tasks were aiming to pick up (e.g. Apraxia and spatial neglect, which are less influenced by education than domain-general cognitive abilities such as memory and executive functioning (e.g. in OCS-plus^47^).

We note that at this point, the Mini-OCS was standardized in the UK English context, providing UK normative cut-offs. In line with previous work with the Oxford Cognitive Screen, we anticipate best practice cultural and language adaptations to be carried out in future research, including generation of additional population-specific normative data.

### Psychometrics

We found strong consistency between time points on Mini-OCS subtasks with no differences found in performance, demonstrating some evidence of test-retest reliability. Evidence of convergent and discriminant validity with standardised neuropsychological test performances above our pre-defined benchmarks demonstrated good construct validity, supporting the Mini-OCS measuring the constructs it is intended to measure in each subtask. Overall, the correlations are in line with typical neuropsychological psychometrics,^15,23,48^ with most large correlations in psychology being around *r* = 0.40.^49^ Few tasks had higher correlations with discriminant tasks than with convergent tasks and where these reversals occurred the convergent correlations were also low. Thus, we interpret the higher discriminant correlations due to error variation with low association rather than true relationships with discriminant variables. Notably, by replacing the domain-general tasks with more sensitive OCS-plus subtasks, the Mini-OCS demonstrated an increased sensitivity to detect impairment in memory and executive function, most relevant in community care.

### Study limitations

Our neurologically healthy control sample was slightly more highly educated than our patient sample, which may subtly limit the interpretation for stroke survivors with low levels of education. Further, our sample was only screened for existing cognitive impairments with the MoCA rather than a full neuropsychological assessment. Assessors relied on self-reports regarding previous neurological and psychiatric problems. Lastly, test–retest reliability was assessed based on a relatively small subsample of mostly controls, as such, potential variance in scores is lost where stroke survivors may change over time. Whilst the present data provide initial insights into the reliability of the Mini-OCS over time, future studies are welcomed to more comprehensively assess test–retest reliability in standardised and clinically relevant intervals.

### Implications for clinical practice

The Mini-OCS was designed as a stroke-specific cognitive screening tool, covering both domain-specific and domain-general cognition, in line with clinical guidelines. Existing tools such as the GPCOG^10^ and 6-Item Cognitive Impairment Test^11^ are language-based and primarily assess memory impairments, characteristic of Alzheimer’s disease and do not assess other cognitive domains (e.g. executive function, praxis, language, number and attention).^50^ Both mentioned tests take 1–3 min less to complete than the Mini-OCS, thus saving clinician time, however, they fail to identify stroke specific cognitive impairments which remain highly common, even long-term post-stroke.^3^ Ideally, the Mini-OCS is used as an initial screen in chronic stroke, where there is a lack of time to screen with OCS and OCS-Plus. If time allows, there is more information present in completing first the OCS and then the OCS-Plus, which would provide a more in-depth screening approach. In either case, following screening, where appropriate, either in order to gain a deeper understanding of the nature of the deficits, or to additionally assess other aspects of cognition, a full neuropsychological assessment referral should be made (see also National Clinical Guideline for Stroke 2023,^50^ on assessment vs Screening).

### Conclusions

The Mini-OCS development was based on a statistically equivalent short-form version of the OCS following item response theory modeling. The Mini-OCS was normed in 164 neurologically healthy adults and validated in a cohort of 89 chronic stroke survivors. The psychometric properties of the Mini-OCS confirm it as a reliable and valid assessment of cognition for use in chronic stroke. Future research should further examine test-retest reliability and the feasibility and practical implementation of using the Mini-OCS in primary care and community settings.

## Supplementary Material

sj-docx-1-eso-23969873251358811
